# The role of vasodilator-stimulated phosphoprotein (VASP) in the control of hepatic gluconeogenic gene expression

**DOI:** 10.1371/journal.pone.0215601

**Published:** 2019-04-24

**Authors:** Sanshiro Tateya, Norma Rizzo-De Leon, Andrew M. Cheng, Brian P. Dick, Woo Je Lee, Madeleine L. Kim, Kevin O’Brien, Gregory J. Morton, Michael W. Schwartz, Francis Kim

**Affiliations:** 1 Department of Medicine, University of Washington, Seattle, WA, United States of America; 2 University of Washington Medicine Diabetes Institute, University of Washington, Seattle, WA, United States of America; Tokyo University of Agriculture, JAPAN

## Abstract

During periods in which glucose absorption from the gastrointestinal (GI) tract is insufficient to meet body requirements, hepatic gluconeogenesis plays a key role to maintain normal blood glucose levels. The current studies investigated the role in this process played by vasodilatory-associated phosphoprotein (VASP), a protein that is phosphorylated in hepatocytes by cAMP/protein kinase A (PKA), a key mediator of the action of glucagon. We report that following stimulation of hepatocytes with 8Br-cAMP, phosphorylation of VASP preceded induction of genes encoding key gluconeogenic enzymes, glucose-6-phosphatase (*G6p*) and phosphoenolpyruvate carboxykinase (*Pck1*), and that VASP overexpression enhanced this gene induction. Conversely, hepatocytes from mice lacking VASP (*Vasp*^*-/-*^*)* displayed blunted induction of gluconeogenic enzymes in response to cAMP, and *Vasp*^*-/-*^ mice exhibited both greater fasting hypoglycemia and blunted hepatic gluconeogenic enzyme gene expression in response to fasting *in vivo*. These effects of VASP deficiency were associated with reduced phosphorylation of both CREB (a key transcription factor for gluconeogenesis that lies downstream of PKA) and histone deacetylase 4 (HDAC4), a combination of effects that inhibit transcription of gluconeogenic genes. These data support a model in which VASP functions as a molecular bridge linking the two key signal transduction pathways governing hepatic gluconeogenic gene expression.

## Introduction

In the fasting state, increased hepatic gluconeogenesis is essential to avert hypoglycemia and maintain normal plasma glucose levels. Glucagon and epinephrine are key hormonal drivers of gluconeogenesis by virtue of increased intracellular cAMP levels induced by binding of their respective receptors on the hepatocyte plasma membrane. This response in turn activates cAMP-dependent protein kinase A (PKA), which ultimately promotes the interaction of cAMP-responsive binding protein (CREB) with CREB-regulated transcription co-activators, resulting in increased expression of genes encoding two key enzymes involved in gluconeogenesis, glucose-6-phosphatase (*G6p)* and phosphoenolpyruvate kinase (*Pck1)* [1, 2].

In addition to this well studied signaling pathway, a distinct histone deacetylase (HDAC)-dependent mechanism has emerged as a participant in the cellular regulation of gluconeogenesis. At the transcriptional level, induction of *G6p* and *Pck1* gene expression requires the interaction of forkhead box o1 (FOXO1) and its co-activator, peroxisome proliferator-activated receptor gamma coactivator-1 alpha (PGC-1α) [3–6], an interaction that is enhanced by deacetylation of both FOXO1 and PGC-1α by the deacetylase, Sirtuin 1 (SIRT1), a class III HDAC [7, 8]. Deacetylation of the transcription factor STAT3 by SIRT1 also enhances gluconeogenesis [9] [10], as does glucagon-induced dephosphorylation of HDAC4,5,7 (class IIa HDAC) resulting in the recruitment of HDAC3 to the nucleus, where it deacytelates and thereby activates FOXO1 [11]. To clarify how PKA signaling is linked to activation of HDACs/SIRT1 signaling, and how these responses are in turn tied to CREB activity [12, 13], the current work focused on the role played by the signaling intermediate vasodilator-stimulated phosphoprotein (VASP).

Discovered in platelets and endothelial cells stimulated with prostaglandins or nitric oxide, VASP belongs to the ENA/VASP family of adaptor proteins linking the cytoskeletal systems to signal transduction pathways. In addition to cytoskeletal organization, fibroblast migration, platelet activation, and axon guidance [14, 15], VASP is also implicated as a mediator of the effect endothelial nitric oxide (NO)/cGMP signaling to attenuates high-fat diet (HFD)-induced insulin resistance and inflammatory activation in hepatic tissue. Conversely, VASP deletion enhances the effect of HFD feeding to increase hepatic NF-κB signaling, thereby impairing hepatic insulin signaling and increasing hepatic triglyceride (TG) content [16]. Together, these findings suggest that during HFD feeding, VASP confers protection against hepatic insulin resistance by inhibiting NF-κB activation. Combined with evidence that increased hepatic steatosis in *Vasp*^*-/-*^ mice results in part from reduced fatty acid oxidation [17], VASP appears to participate in mechanisms that link dietary fat content to hepatic insulin action. Relevant to the current work is the key physiological role played by insulin to inhibit hepatic gluconeogenesis through activation of FOXO1 [11]. Indeed, the effect of fasting to inhibit insulin secretion is required for the associated increase of gluconeogenesis.

VASP has three primary phosphorylation sites: *1)* Serine 239, which is phosphorylated primarily by PKG; *2)* Serine 157, which is phosphorylated by both PKA and PKC; and *3)* Threonine 278, which is phosphorylated by AMP-activated protein kinase (AMPK) [18]. Since the cAMP/PKA pathway is a crucial downstream mediator of glucagon receptor signaling, we hypothesized a role for activation of VASP (by PKA-mediated phosphorylation of Serine 157) in the effect of fasting to increase hepatic gluconeogenesis. We further hypothesized that the effects of VASP on gluconeogenesis involve phosphorylation of downstream targets including both SIRT1/HDACs and CREB.

## Materials and methods

### Animal experiments

Eight week old *WT (C57Bl/6*)(Jackson Laboratory) and *Vasp*^*-/-*^ mice [16] were maintained on a low-fat (LF; 10% saturated fat, D12492; Research Diets, New Brunswick, NJ) for 4 wk. To evaluate hepatic gluconeogenesis responses *in vivo*, glucagon (1μg) or sodium pyruvate (2g/kg) was injected (IP) after a 6 h fast and at the end of the experiment, both blood and liver samples were collected. In all experiments, age-matched groups of adult mice were used. All animals were maintained in a temperature-controlled facility with a 12-hour light-dark (06:00–18:00) cycle and with free access to water and chow. We defined fasting as a state where the mice do not have access to food but remain to have ad libitum access to water. For longer-term, overnight fasting studies, food was removed from mice early/mid dark cycle (22:00) for a period of either 12h or 16 h, with tissue and blood collection at 10:00 and 14:00, respectively. For short-term fasting periods (1–4 h) food was removed early/mid light cycle at 10:00, with tissue and blood collection between 11:00 and 14:00. All procedures were approved by the University of Washington Institutional Animal Care and Use Committee and all experiments were performed in accordance with relevant guidelines and regulations. Animals were reviewed daily by veterinary technicians and research team. Animal death was not an endpoint of the study. For sacrifice, animals were deeply anesthetized using isoflurane until the hind toe pinch is ablated before decapitation. Blood (700–1000 μl) was obtained via cardiac puncture and liver tissue was snap frozen in liquid nitrogen.

### Quantitative RT-PCR analyses

RNA was extracted using RNAase kit (Quiagen; Valencia, CA). For gene expression analysis, real-time RT-PCR reactions were conducted as described previously [16]using TaqMan Gene Expression Analysis (Applied Biosystems; Foster City, CA).

### Western blotting

Cell lysis and tissue extraction were performed as described previously [16]. All Western blots used equal amounts of total protein for each condition from individual experiments, and were performed as described previously [16]. For immunoprecipitation experiments, lysates were centrifuged for 5 min at 14,000 rpm and equivalent amounts of protein as determined by Bradford assay were used for immunoprecipitations. The supernatants were precleared and incubated at 4°C overnight with 10–20 μl of anti-acetyl-Lysine antibody with 60 μl Protein A/G agarose suspension.

## Materials

Anti-VASP(#3132), anti-phosphorylated VASP (Ser157)(#3111), anti-phosphorylated serum response factor (serine103) (SRF)(#4261) and total SRF(#5147) antibodies were purchased from Cell Signaling (Denvers, MA). Anti-GAPDH (sc-25778) rabbit polyclonal antibody and anti CREB binding protein (CBP) (sc369) were obtained from Santa Cruz Biotechnology (Santa Cruz, CA).

### Cell culture

AML12 hepatocytes were purchased from American Type Culture Collection (Manassas, VA) and cultured in DMEM/F-12 50/50 (10-090-CV, Cellgro) with 3.151mg/ml D-glucose, 0.005mg/ml insulin, 0.005mg/ml transferrin, 5ng/ml selenium, 40ng/ml dexamethasone, and 10% FBS as described previously [16].

A retroviral construct containing human wild-type VASP in the retroviral vector LXSN [16] was used for overexpression studies. AML12 cells were infected with LXSN or LXSN-wt-VASP virus. Multiple clones were selected, propagated, and maintained in the presence of G418 (0.6 mg/ml, Cellgro; Manassas, VA). AML12 hepatocytes were chosen for the VASP overexpression studies since VASP protein was reproducibly overexpressed over 3–4 cell passages.

Primary hepatocyte cell culture was performed as described previously [16, 17]. In brief, after hepatic perfusion through the portal vein with liver digest medium (GIBCO) containing 0.05mg/ml of collagenese type 4 (Worthington Biochemical Corporation, Lakewood, NJ), the liver was minced and transferred into a 50ml conical tube through a 70 μm cell strainer. After centrifugation (5 min at 50 g) primary hepatocytes (the cell pellet) were collected and cultured in a same media as for AML12 cells. In vitro experiments to examine gluconeogenesis were performed after deprivation of serum for 16 hours [4].

### Knockdown of *Creb1* and *Srf* by siRNA in AML12 cells

AML12 hepatocytes were transfected with either a scrambled siRNA (4390843, Ambion, Austin, TX) or siRNA directed against *Creb* (*creb1*; s232195, Ambion, Austin, TX) or *Srf* (*srf*; s74390, Ambion, Austin, TX) using siPORT NeoFX Transfection Agent (Ambion, Austin, TX) according to the manufacturer’s protocol.

### Statistical analysis

In all experiments, densitometry measurements were normalized to controls incubated with vehicle and fold increase above the control condition was calculated. Analysis of the results was performed using the Graphpad statistical package. Data are expressed as means ± SEM, and values of *p*<0.05 were considered statistically significant. A two-tailed *t* test was used to compare mean values in two-group comparisons. For four-group comparisons, two-way ANOVA, and the Bonferoni post hoc comparison test were used to compare mean values between groups.

## Results

### Time course of the effect of fasting on hepatic VASP ser157 phosphorylation

**Fig 1:**
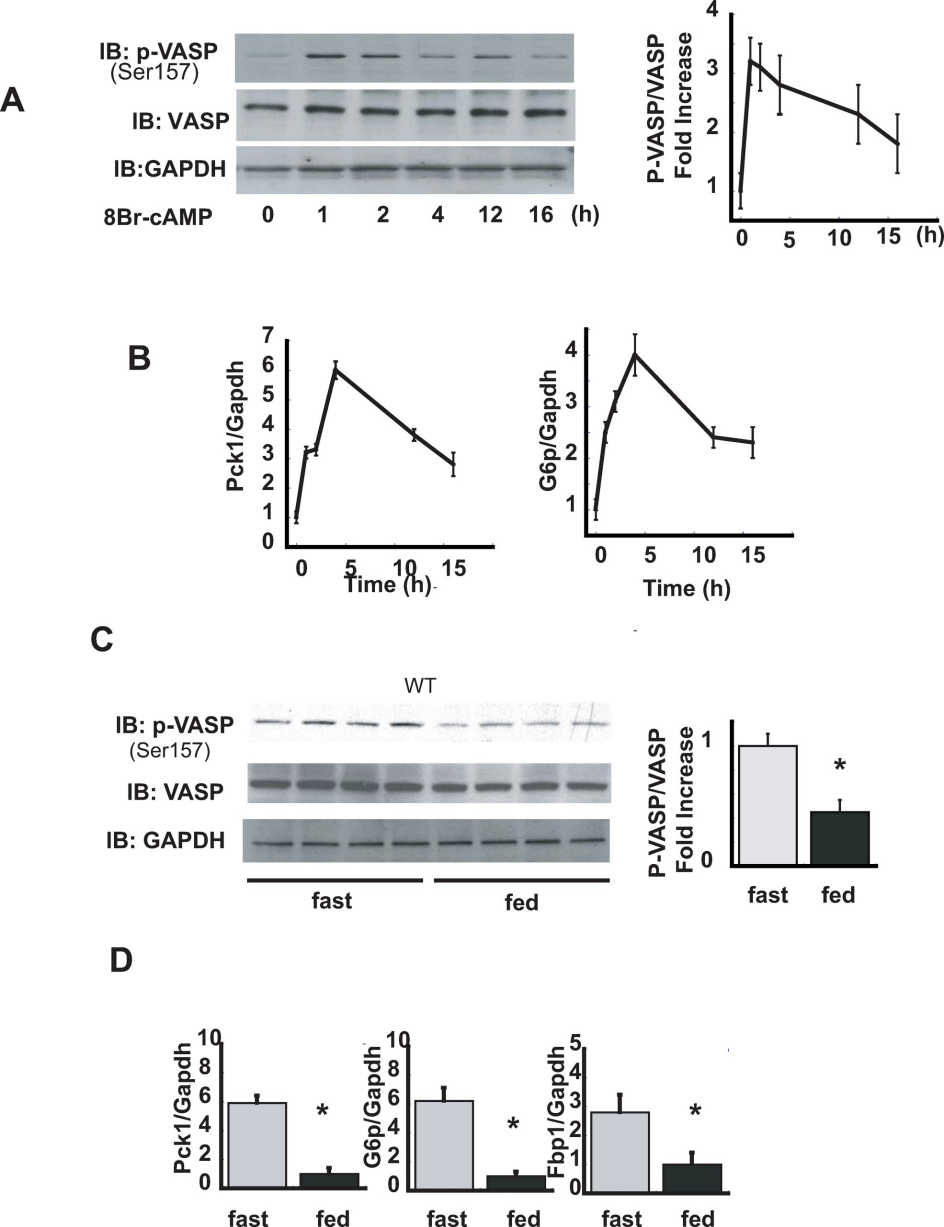
The effect of 8Br-cAMP and fasting on hepatic VASP (Ser157 phosphorylation) levels. **A**. AML12 hepatocytes were treated with 8Br-cAMP (100μM) for a time period between 0–16 h, and both protein and RNA was harvested. Protein lysates were analyzed for p-VASP (Ser157) and VASP protein levels by Western blot (n = 3) p<0.05 time 0 vs. 1 h. **B.***Pck1* and *G6p* levels were determined by RT-PCR. Relative mRNA levels were normalized to *Gapdh* (n = 3) p<0.05 time 0 vs. 4h. **C**. C57BL6 male mice on chow diet were sacrificed either in the fed state or after 4 hours fast. Hepatic expression of p-VASP (Ser157) as measured by Western blot (n = 4 fast, n = 4 fed). **D**.*Pck1*,*G6p*, *Fbp1* by RT-PCR (n = 4 fast, n = 4 fed). *P<0.05 *WT*, wild type; IB, immunoblot.

During fasting, the combination of reduced plasma insulin and increased plasma glucagon levels supports the maintenance of normal blood glucose levels by inducing hepatic gluconeogenesis via a mechanism involving both reduced hepatocyte FOXO1 and increased cAMP/PKA signaling, leading to *Pck1 and G6p* gene expression [1, 2]. Since PKA phosphorylates VASP on serine157 in other cell types [18, 19], we first tested whether cAMP stimulation increases VASP ser157 phosphorylation in hepatocytes, and if so, whether this occurs over a time course consistent with a role in gluconeogenesis (e.g., prior to the induction of *Pck1* or *G6p*). AML12 hepatocytes were treated with 8Br-cAMP (100μM) for 1–16 h and lysates analyzed by both Western blot and RT-PCR. As expected, VASP phosphorylation on serine 157 was increased by 3- fold over controls in response to cAMP stimulation by 8-bromo-cAMP, peaking 1 hour after treatment (Fig 1A), well before the increase of *Pck1* and *G6p* gene expression (Fig 1B).

We next sought to determine if fasting recapitulates this effect *in vivo*. Compared to the fed state, mice subjected to a 4 h fast displayed a 2-fold increase of hepatic p-VASP (Ser157) content (Fig 1C), an effect associated with 6-fold increases of both *Pck1*, *G6p Fbp1* gene expression (Fig 1D). These observations are consistent with, but do not directly test, the hypothesis that VASP activation participates in the effect of fasting to increase hepatic *Pck1* and *G6p* gene expression.

### Role of VASP in hepatocyte PEPCK and G6P expression

**Fig 2:**
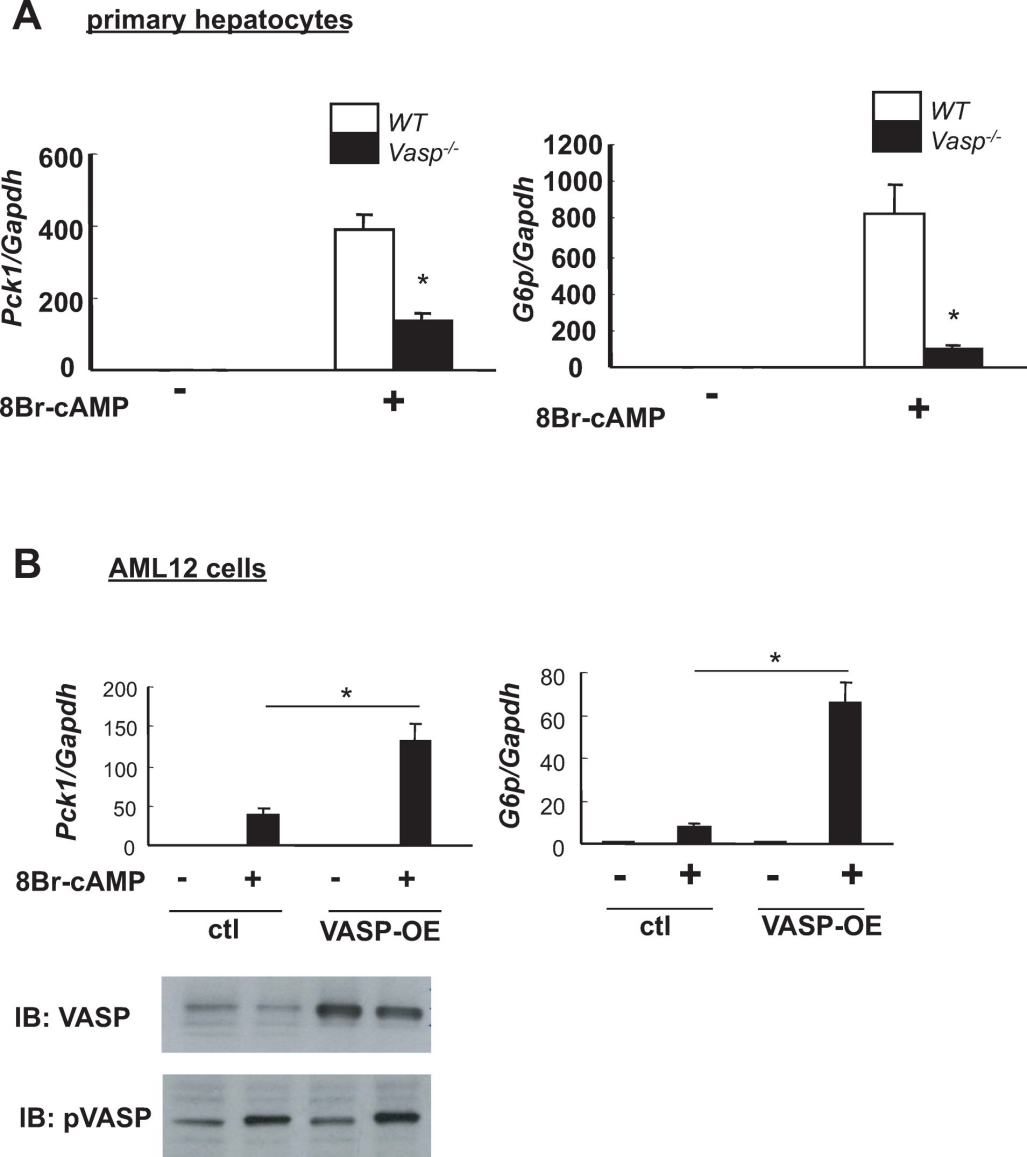
The effect of VASP on PCK1 and G6P in hepatocytes. **A**. Primary hepatocytes were isolated from *WT* or *Vasp*^*-/-*^ mice and stimulated with 8Br-cAMP (100μM, 4 hours). Relative mRNA levels of *Pck1* and *G6p* as measured by RT-PCR (n = 5 WT, n = 5 Vasp-/-). **B**. AML12 hepatocytes were transduced with VASP (VASP-OE) or empty (ctl) vector and were treated with 8Br-cAMP (100μM, 4 hours). Relative mRNA expressions of *Pck1* and *G6p* as measured by RT-PCR (n = 5). Total VASP and phospho-VASP protein as assessed by Western blot. *P<0.05 *WT*, wild type; IB, immunoblot.

To determine if VASP is either necessary or sufficient for cAMP-mediated induction of gluconeogenic gene expression in hepatocytes, we used *1)* primary hepatocytes derived from WT and *Vasp*^*-/-*^ mice, and *2)* AML12 hepatocytes in which VASP was overexpressed using a retroviral vector [16], respectively. Compared to control hepatocytes from WT mice, cAMP-mediated induction of gluconeogenic enzymes was markedly attenuated in *Vasp*^*-/-*^ hepatocytes (Fig 2A), whereas in hepatocytes in which VASP is overexpressed, cAMP-mediated induction of *Pck1* and *G6p* was increased dramatically compared to the hepatocytes transduced with control vector (Fig 2B). In contrast, basal levels (e.g., in the absence of 8 Br-cAMP stimulation) *Pck1* and *G6p* mRNA levels were unaffected by either knockout or overexpression of VASP. Thus, VASP appears to be both necessary and sufficient to mediate cAMP-induced gluconeogenic gene expression in hepatocytes. A fundamental question raised by these observations is whether intact VASP signaling is also required for glucose homeostasis *in vivo*.

### The effect of fasting on blood glucose levels in *Vasp*^*-/-*^ mice

**Fig 3:**
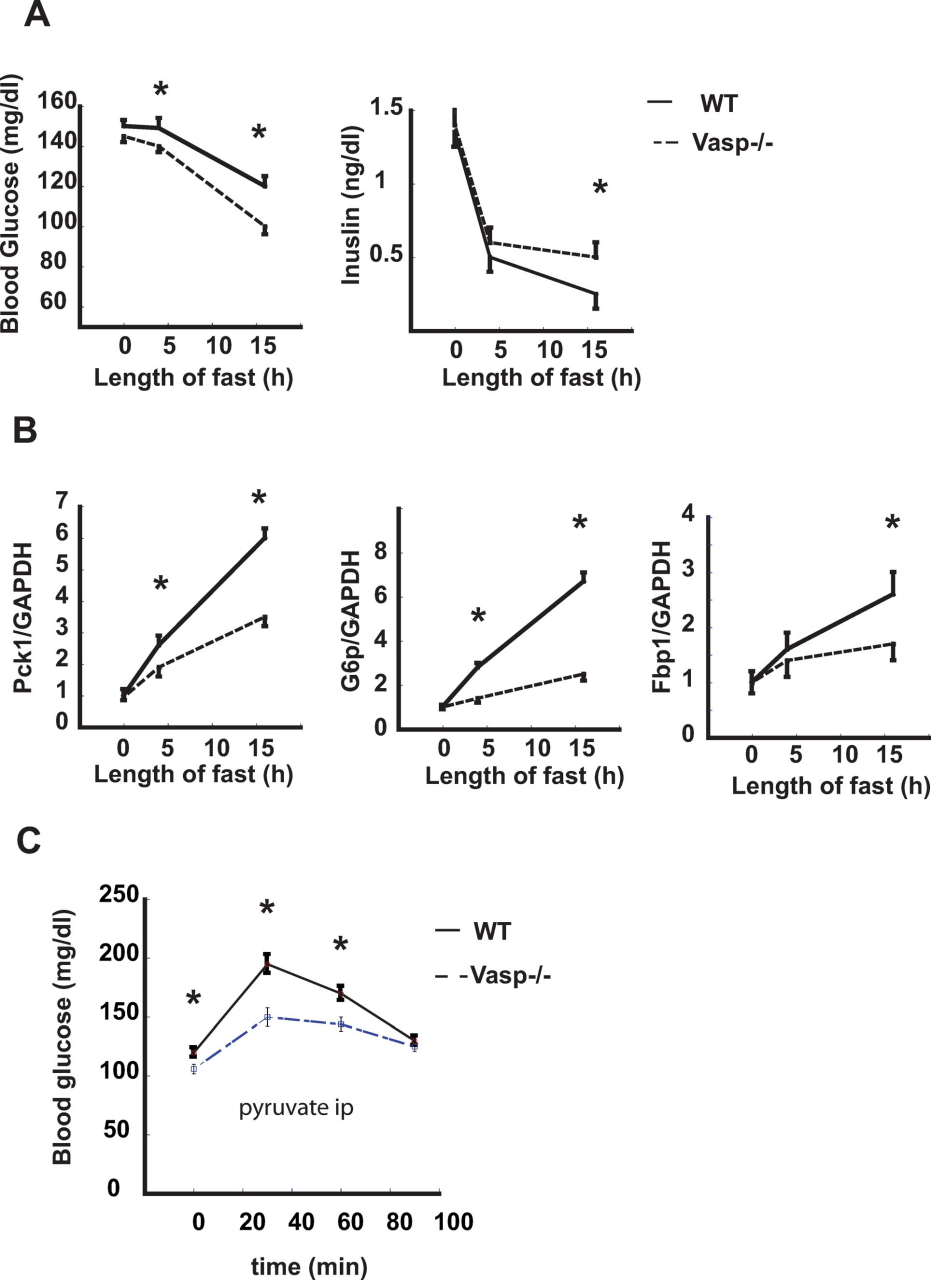
The effect of fasting on glucose level in *Vasp*^*-/-*^ mice. **A.** Blood glucose levels and insulin levels from baseline during starvation in *WT* and *Vasp*^*-/-*^ mice (n = 7). **B**. Relative mRNA expression of *Pck1* and *G6p* as measured by RT-PCR during fasting period (n = 7). **C**. After a 6 h fast, sodium pyruvate (2g/kg) was injected (IP) in *WT* and *Vasp*^*-/-*^ mice, followed by serial measurements of blood glucose level (n = 7). *P<0.05 *WT*, wild type; ip- intraperitoneal.

To determine if VASP is required for normal glucose homeostasis *in vivo*, we monitored levels of both blood glucose and *Pck1*, *G6p and Fbp1* expression after fasting for 0 h, 4 h or 16 h in *WT* and *Vasp*^*-/-*^ mice respectively. Whereas body weight and fed glucose levels did not differ between WT (25.1 ± 0.8 g) and *Vasp*^*-/-*^ mice (26 ±0.9 g) (p = not significant) blood glucose levels were significantly lower in *Vasp*^*-/-*^ mice at both the 4 –h and 16-h time point (Fig 3A), and the absolute change of blood glucose from the fed level to the 16-h time point also achieved statistical significance. Thus, the effect of fasting to lower blood glucose levels was exaggerated in *Vasp*^*-/-*^ than in *WT* mice. Conversely, fasting insulin levels were higher at the 16-h time point (Fig 3A). Consistent with this finding, fasting for 4 or 16 h also increased hepatic levels of both *G6p*, *Pck*, *Fbp1* mRNA in a time-dependent manner, and this effect was blunted by ~50% in *Vasp*^*-/-*^ compared to WT mice (Fig 3B).

Since pyruvate is converted to glucose during gluconeogenesis, the pyruvate tolerance test can be used to assess the integrity of this process *in vivo*. We therefore subjected both WT and *Vasp*^*-/-*^ mice to a pyruvate tolerance test and, as expected, IP injection of pyruvate (2g/kg) increased glucose levels over the 90-min study period in both genotypes. However, the glucose excursion induced by pyruvate administration was attenuated in *Vasp*^*-/-*^ compared to *WT* mice (Fig 3C), offering further evidence that hepatic gluconeogenesis is impaired by VASP deficiency.

### VASP enhances gluconeogenesis through CREB

**Fig 4:**
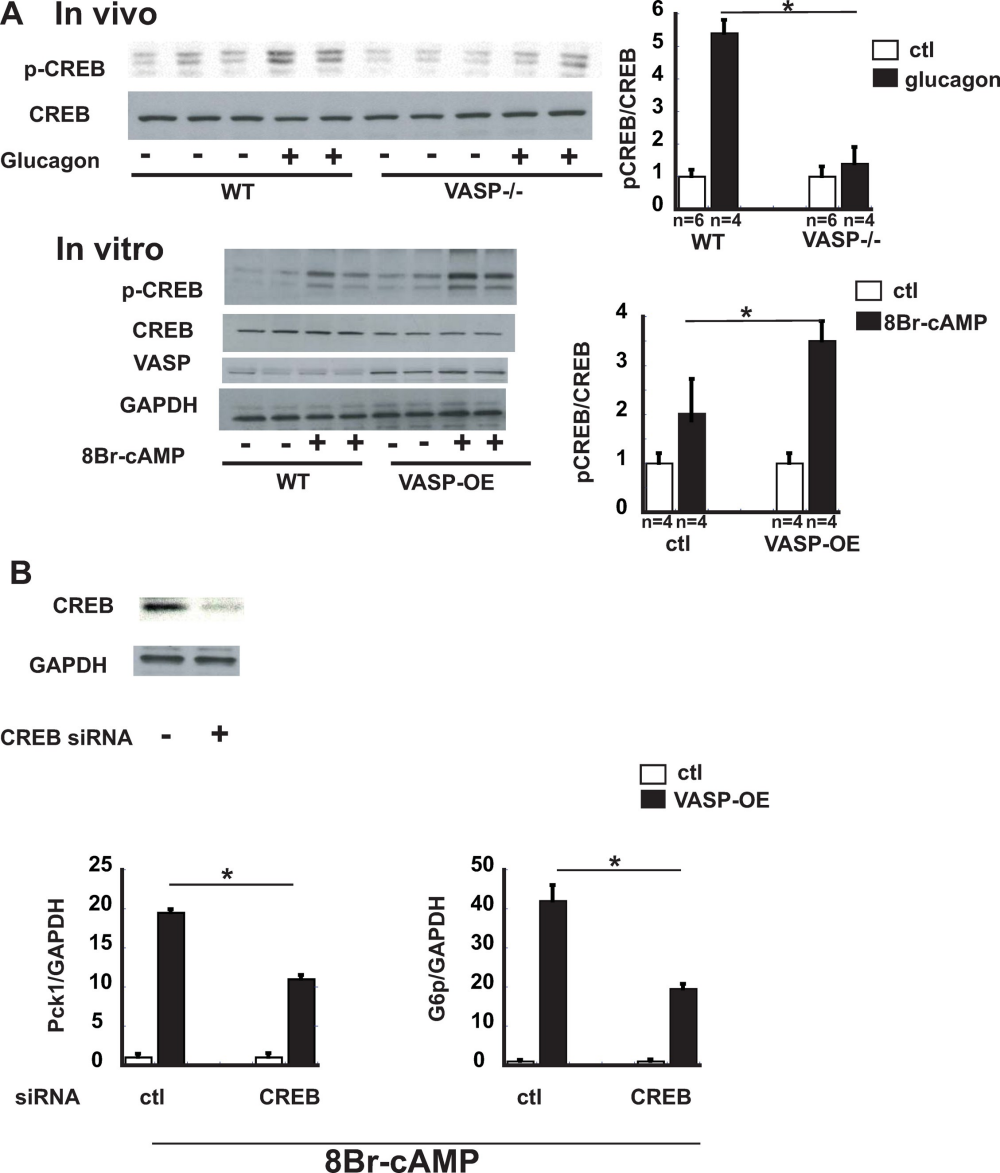
VASP enhances gluconeogenesis through CREB. **A.** (upper panel) Following a 6 hour fast, *WT (n = 4)* or *Vasp*^*-/-*^ mice (n = 4) received IP glucagon (1μg). Mice were sacrificed 30 min later and liver tissues were collected. Phospho-CREB and total CREB levels were assessed by Western blot. (lower panel). AML12 hepatocytes transduced with VASP were stimulated with 8Br-cAMP (100μM) for 30 min, followed by Western blot analysis for p-CREB and total CREB. **B.** (upper panel) Western blot of CREB following siRNA for CREB (creb1, 5nM, 24h) in AML12 hepatocytes. (lower panel) Cells were treated with 8Br-cAMP (100μM, 4h) and RNA was extracted. RT-PCR analysis of *Pck1* and *G6p* treated with siRNA for CREB in VASP-overexpressed AML12 hepatocytes. (n = 5). *P<0.05 *WT*, wild type; IB, immunoblot.

During fasting, phosphorylation of the transcription factor CREB is required for the effect of cAMP/PKA signaling to induce gluconeogenic enzyme gene expression in hepatocytes. Based on our evidence that the latter effect is also dependent on VASP (Fig 2A), we hypothesized that VASP lies downstream of PKA in the mechanism leading to enhanced CREB-mediated transcriptional activity. As an initial test of this hypothesis, we administered glucagon (1μg IP) to both *Vasp*^*-/-*^ and control WT mice and after sacrifice, measured phospho-CREB levels by Western blot analysis of liver lysates. Compared to WT mice, glucagon-stimulated CREB phosphorylation was dramatically reduced in the liver of *Vasp*^*-/-*^ mice (Fig 4A). Conversely, overexpression of VASP in AML12 hepatocytes increased basal p-CREB levels and also dramatically enhanced the effect of 8Br-cAMP to increase CREB phosphorylation (Fig 4A).

Since VASP overexpression enhances both CREB phosphorylation (Fig 4A) and cAMP-mediated induction of *Pck1* and *G6p* in AML12 hepatocytes (Fig 2B), we hypothesized that VASP stimulates gluconeogenic gene expression via a CREB-dependent mechanism. To test this hypothesis, we investigated the effect of VASP overexpression after CREB protein knockdown in these cells, an effect known to blunt cAMP/PKA induction of gluconeogenesis (3). Consistent with this hypothesis, reducing CREB expression in AML12 hepatocytes (by exposing them to *Creb* siRNA; Fig 4B) diminished the effect of VASP overexpression to enhance *Pck1* and *G6p* induction (Fig 4B). These data identify CREB, a key regulator of gluconeogenesis, in the mechanism linking VASP to gluconeogenic gene induction in hepatocytes.

### The effect of VASP on serum response factor during induction of gluconeogenesis

**Fig 5:**
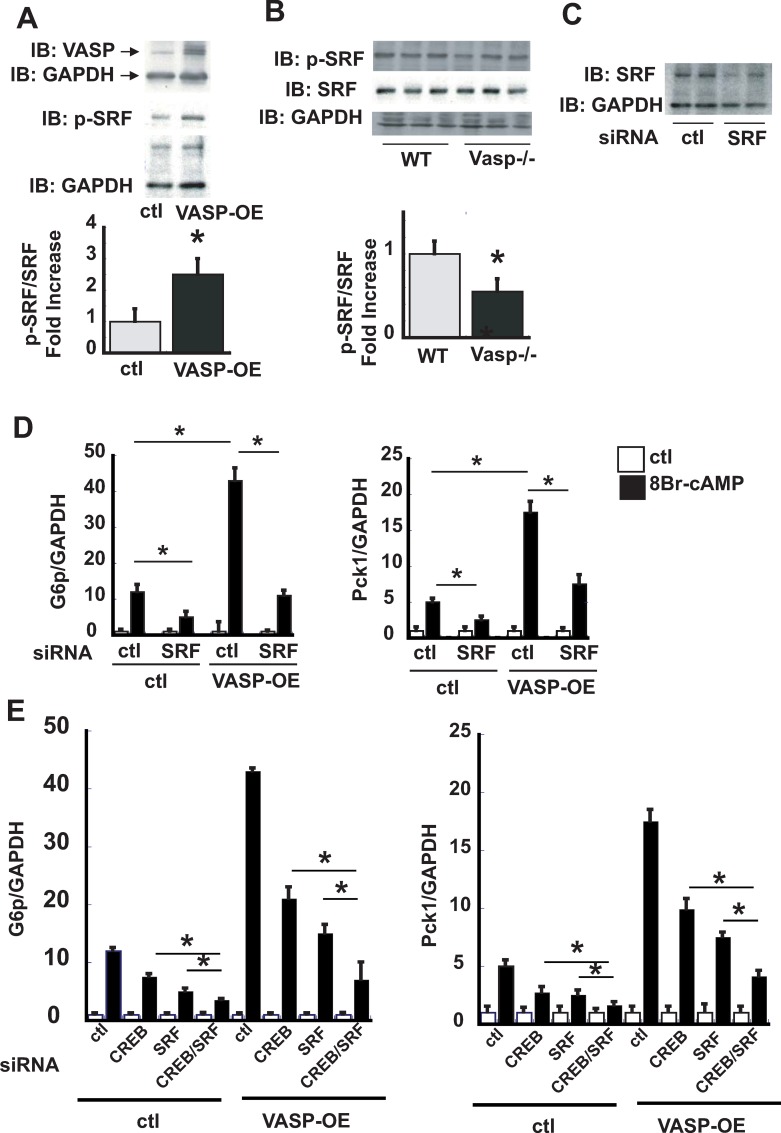
The effect of VASP on serum response factor during induction of gluconeogenesis. Phospho-SRF as measured by Western blot in AML12 hepatocytes overexpressing VASP **A** (n = 4). or in primary hepatocytes isolated from *WT* or *Vasp*^*-/-*^ mice **B.** WT (n = 3), Vasp-/- (n = 3) **C**. SRF protein levels following siRNA for SRF (srf, 5nM, 24h) as assessed by Western blot. **D.** RT-PCR analysis of *Pck1* and *G6p following treatment with* 8Br-cAMP (100μM, 4 hours) *in* empty vector-transduced or VASP-overexpressed AML12 hepatoctyes, *in the presence or absence of* siRNA to SRF (n = 4). **E**. RT-PCR analysis of *Pck1* and *G6p* in response to 8Br-cAMP(100μM, 4 hours) in the presence of siRNA to SRF or to CREB in empty vector-transduced or VASP-overexpressed AML12 hepatocytes. (n = 5). Representative blots are shown. *P<0.05 *WT*, wild type; IB, immunoblot; IP, immunoprecipitation.

Serum response factor (SRF) is a transcription factor implicated in regulating liver metabolism and a known nuclear target of VASP [20, 21]. Since mice with liver specific-SRF knockout display fasting hypoglycemia [21], we hypothesized that the stimulatory effect of VASP on gluconeogenic gene expression may involve activation of SRF as well as CREB. Consistent with this hypothesis, VASP overexpression increased p-SRF levels in AML12 cells (Fig 5A), whereas in liver lysates isolated from *Vasp*^*-/-*^ mice after an overnight fast, p-SRF levels were reduced relative to those of WT controls (Fig 5B). Thus, VASP overexpression and VASP knockout increase and decrease hepatic p-SRF content, respectively, suggesting a key role for VASP signaling to regulate SRF activity in hepatocytes.

To determine if, like CREB, SRF is required for the stimulatory effect of VASP on gluconeogenic gene expression, we studied AML12 hepatocytes after reducing SRF protein levels by siRNA (Fig 5C). As predicted, this intervention attenuated the induction of *Pck1* and *G6p* by VASP overexpression (Fig 5D), suggesting that SRF is required for VASP-mediated effects on gluconeogenesis. Furthermore, combined knockdown of both SRF and CREB decreased the response to 8Br-cAMP (measured as the increase of cAMP-mediated *Pck1* and *G6p* expression) to a greater extent than did knockdown of SRF or CREB alone (Fig 5E). These results collectively implicate SRF and CREB as distinct downstream targets of VASP signaling that serve to link increased glucoeneogenesis to stimulation by cAMP.

### The effect of VASP on HDACs/SIRT1 dependent regulation of gluconeogenesis

In addition to the cAMP-PKA-CREB pathway, transcriptional control of gluconeogenic enzyme induction is regulated by the acetylation status of key transcription factors. Specifically, the activity of PGC1α, FOXO1 and STAT3, each of which binds to the promoter region of *Pck1*, *G6p* and other target genes [6–8, 10, 22, 23], is reduced by acetylation, and therefore can be increased by deacetylation. SIRT1 is a class III HDAC that increases gluconeogenesis by deacetylating PGC1α, FOXO1 and STAT3. The observation that endothelially-derived nitric oxide, an upstream activator of VASP [16], also activates SIRT1 (26), suggests that VASP might increase gluconeogenesis in part by activating SIRT1. Similarly, HDAC4, which is known to deacetlylate FOXO1 and thereby promote transcription of genes encoding *Pck1* and *G6p [11, 12]*, is dephosphorylated following cAMP stimulation. This effect in turn causes HDAC4,5,7 to recruit HDAC3 to be translocated to the nucleus where, like SIRT1, it increases FOXO1 transcriptional activity.

**Fig 6:**
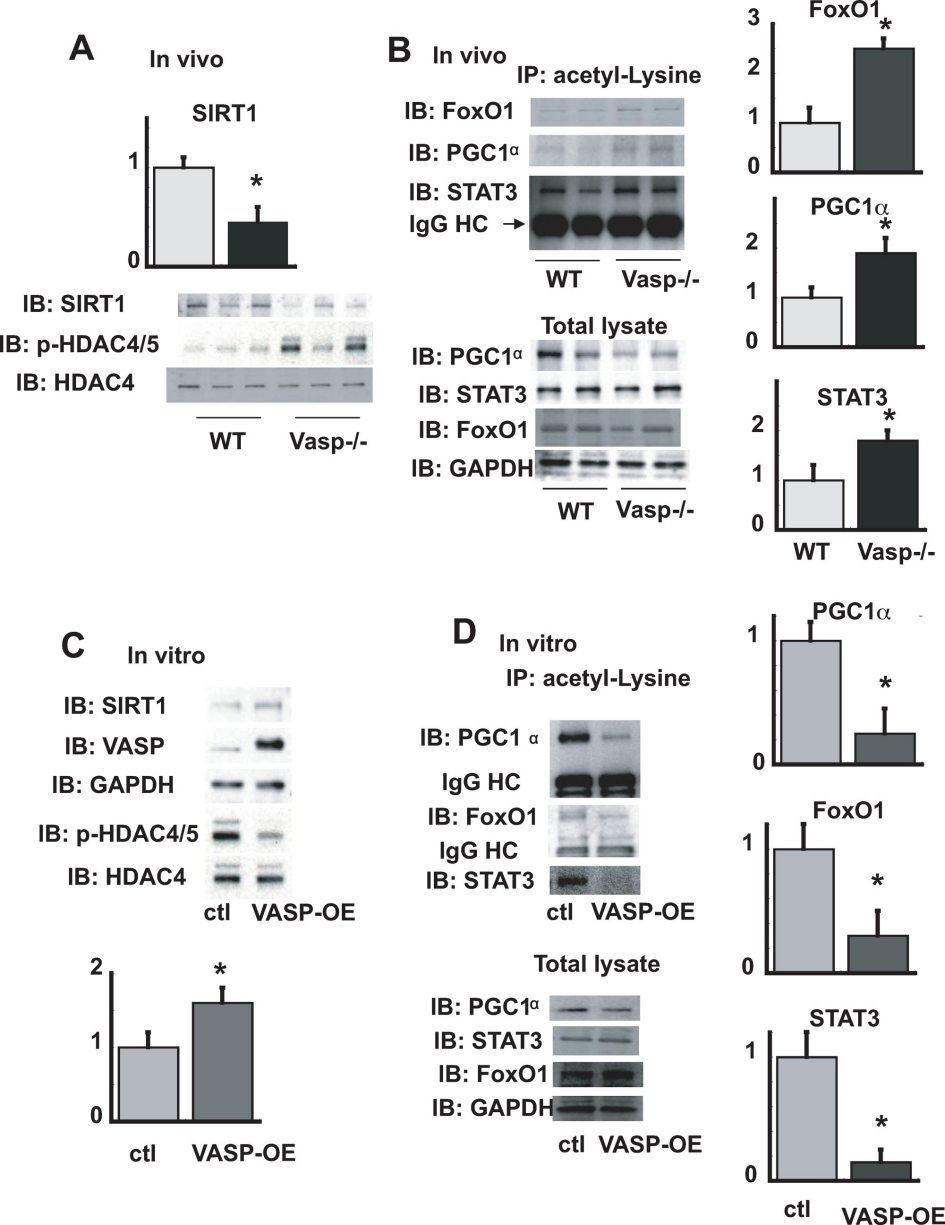
The effect of VASP on HDACs/SIRT1 dependent regulation of gluconeogenesis. *WT* or *Vasp*^*-/-*^ mice were sacrificed after an overnight fast and liver lysates were made. **A**. Western blot analysis for SIRT1, p-HDAC4/5, and HDAC4 (n = 4 WT, n = 4 Vasp -/-). **B.**Western blot analysis with anti-FoxO1, anti-PGC1α, anti STAT3 antibodies following immunoprecipitation of liver lystates with anti-acetyl-Lysine antibody(n = 4 WT, n = 4 Vasp-/-). **C**.Western blot of VASP overexpression or control AML12 hepatocytes using anti- SIRT1, VASP, GAPHDH, p-HDAC4, HDAC4 antibodies. **D**. Western blot analysis with indicated antibodies following immunoprecipitation with anti-acetyl-Lysine antibody in lysates from control or VASP overexpressing AML12 hepatocytes. Representative blots are shown (n = 4 ctl, n = 4 VASP OE). *P<0.05 *WT*, wild type; IB, immunoblot; IP, immunoprecipitation.

To determine if actions of VASP involve SIRT1, HDAC4, and subsequent deacetylation of FOXO1, PGC1α, and STAT3, we first studied hepatocytes derived from *Vasp*^*-/-*^ mice. Compared to hepatocytes from WT mice, expression of SIRT1 and p-HDAC4/5 were reduced and increased, respectively (Fig 6A), consistent with reduced activity of both deacetylases. Moreover, these effects were accompanied by increases in acetylation of their substrates, FOXO1, PGC1α, and STAT3 (Fig 6B), which reduces their transcriptional activity. These observations are consistent with our earlier findings of both reduced expression of gluconeogenic genes and augmentation of the effect of fasting to lower blood glucose levels in these animals. Conversely, overexpression of VASP in AML12 cells had the opposite effect, increasing both SIRT1 expression and dephosphorylation of HDAC4 (Fig 6C), coincident with reductions in the acetylation of their substrates (Fig 6D). These data suggest that VASP enhances SIRT1/HDAC4 signaling and thereby deacetylates FOXO1, PGC1α, and STAT3, increasing their transcriptional activity and thereby enabling intact gluconeogenesis during fasting.

## Discussion

Glucose homeostasis is achieved through a highly coordinated regulatory system that governs both glucose production (occuring primarily in liver) and utilization and, in normal individuals, maintains blood glucose within narrow physiological limits. Control of gluconeogenesis is a key element of this regulatory system, and dysregulation of this process contributes to hyperglycemia in patients with diabetes [24]. In the fed state, gluconeogenesis is inhibited both by reduced secretion of glucagon [24] and through the suppressive effects of insulin. Conversely, increased hepatic glucose production during a fast is crucial for maintenance of euglycemia, a process that over time becomes increasingly dependent on gluconeogenesis [25]. Our new findings support a novel role for VASP, acting via two distinct hepatocyte signal transduction pathways, in this process.

In addition to the classical cAMP/CREB pathway, induction of gluconeogenic genes involves deacetylation (and thereby activation) of 3 key transcription factors—PGC1α, FOXO1, and STAT3 [6–11], but how these two regulatory pathways are linked to one another remains uncertain [11–13]. Our data offers evidence that VASP serves a molecular bridge linking the classical cAMP/CREB pathway to deacetylation-mediated via SIRT1 and HDAC4. In support of this model, we found not only that VASP is required for the ability of PKA to activate CREB and thereby induce *Pck1* and *G6p* expression in liver, but that VASP overexpression activates both SIRT1 and HDAC4, resulting in deacetylation (and hence activation) PGC1α, FOXO1, and STAT3.

Our data also reveal a connection between VASP and regulation of *Pck1 and G6p* not only by CREB, but also by SRF, a ubiquitously expressed transcription factor that binds to a DNA *cis* element known as the CArG box[26]. CREB stimulates a number of growth factor- and stress-responsive genes by forming a complex involving SRF [27, 28] along with Creb binding protein (CBP), a co-activator for CREB [29–32]. Our data suggest that VASP enhances the formation of this transcriptional complex, as cAMP-stimulated phosphorylation of both SRF and CREB is increased by VASP overexpression and blunted by VASP deficiency.

We previously reported that VASP also lies downstream of PKG in a pathway activated by nitric oxide (NO) released from endothelial cells, and that VASP activation via this mechanism attenuates high-fat (HF)-mediated insulin resistance and inflammatory activation in hepatic tissue. Since the absence of VASP enhances NF-κB signaling in the liver, impairs hepatic insulin signaling and increases hepatic triglyceride content during HFD feeding [16], activity of this enzyme appears to be required to prevent inflammatory activation and insulin resistance in hepatocytes. Accordingly, we anticipated that both gluconeogenesis and blood glucose levels would also be increased by VASP deficiency but, in sharp contrast to this expectation, we report here that *Vasp-/-* mice exhibit impaired gluconeogenesis and an exaggerated decline of blood glucose levels during a fast.

These seemingly paradoxical effects are best explained by a dual role for VASP in hepatic metabolism. Specifically, the effect of VASP to reduce inflammation, fatty acid oxidation and insulin action on the one hand, while increasing gluconeogenesis on the other, likely reflects distinct roles for VASP in the control of cellular inflammation and gluconeogenesis. In this context, VASP shares a striking similarity to effects of SIRT1 in liver metabolism. The finding that SIRT1 activation stimulates FFA oxidation, reduces cellular inflammation, and promotes a lean, insulin-sensitive phenotype [33–35] has identified it as a molecular target for diabetes drug development. Yet SIRT1 also participates in the transcriptional control of gluconeogenesis and is required for intact hepatic responses to fasting.

Our finding that VASP regulates SIRT1 activity (Figs 6A, C) offers evidence of a functional link between the paradoxical effects of these two enzymes on hepatic metabolism. Although the basis for these effects remains uncertain, several possibilities can be considered. In the case of VASP, we favor the possibility that its distinct biological functions are mediated by phosphorylation of distinct serine residues; *i*.*e*., that VASP activation triggers distinct cellular responses depending on which serine is phosphorylated. Consistent with this model, PKA-mediated activation of VASP (which promotes gluconeogenesis) involves phosphorylation of Ser 157, whereas the anti-inflammatory, insulin sensitizing effects of VASP appear to involve phosphorylation of Ser239 via the NO/cGMP/PKG pathway. Part of the appeal of this model is its potential to explain the opposing effects of hepatic signaling by PKA and PKG in the control of hepatic metabolism–mediated via distinct effects on VASP activity—and additional studies are warranted to test this hypothesis.

## Conclusions

In summary, we provide evidence that in the liver, VASP phosphorylation contributes to activation of the CREB transcriptional complex required for stimulation of Pck1, G6p, and provides a molecular bridge linking CREB to SIRT1/HDAC4 signaling in the control of this process.

## Supporting information

S1 Fig Supporting information for Fig 1. (PDF)

S2 Fig Supporting information for Fig 2. (PDF)

S3 Fig Supporting information for Fig 4. (PDF)

S4 Fig Supporting information for Fig 6. (PDF)
