# Supplementary figures and images for: The role of vasodilator-stimulated phosphoprotein (VASP) in the control of hepatic gluconeogenic gene expression
Source: PLoS One. 2019 Apr 24;14(4):e0215601. doi: 10.1371/journal.pone.0215601 (PMC6481847; doi:10.1371/journal.pone.0215601)

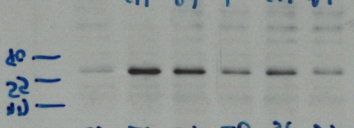

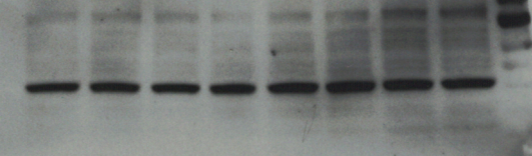

10

22

10

22

10

22

10

22

10

22

10

22

10

22

10

22

10

22

10

22

10

22

10

22

10

22

10

22

10

22

10

22

10

22

10

22

10

22

10

22

10

22

10

22

10

22

10

22

10

22

10

22

10

22

10

22

10

22

10

22

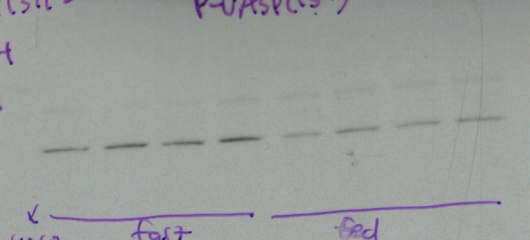

Supplement: S1 Fig — (PDF) [file pone.0215601.s001.pdf]

—

—

—

—

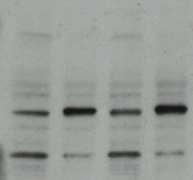

Supplement: S2 Fig — (PDF) [file pone.0215601.s002.pdf]

==

==

==

==

==

==

==

==

==

==

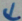

pCREB

113112

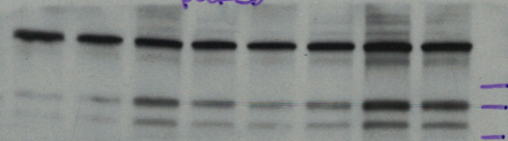

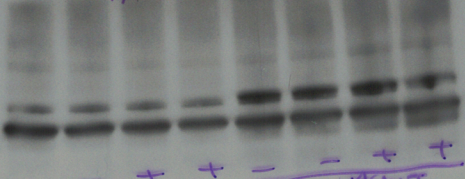

Supplement: S3 Fig — (PDF) [file pone.0215601.s003.pdf]

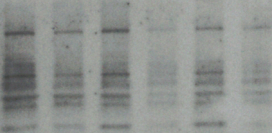

\_\_\_\_\_

\_\_\_\_\_

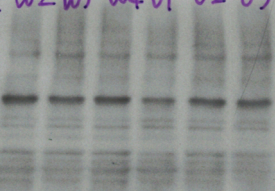

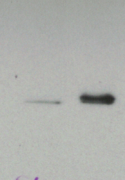

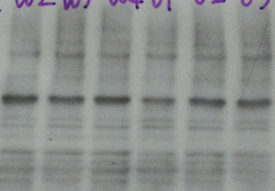

+ + - -

---

W

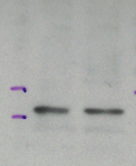

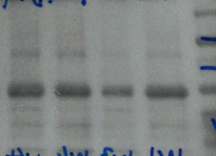

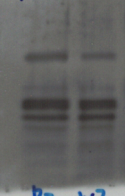

100  
70

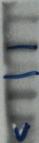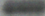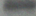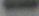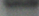

Supplement: S4 Fig — (PDF) [file pone.0215601.s004.pdf]
